# Attribute Selection Based on Constraint Gain and Depth Optimal for a Decision Tree

**DOI:** 10.3390/e21020198

**Published:** 2019-02-19

**Authors:** Huaining Sun, Xuegang Hu, Yuhong Zhang

**Affiliations:** 1 School of Computer Science, Huainan Normal University, Huainan 232038, China; 2 School of Computer and Information, Hefei University and Technology, Hefei 230009, China

**Keywords:** decision tree, attribute selection measure, entropy, constraint entropy, constraint gain, branch convergence and fan-out

## Abstract

Uncertainty evaluation based on statistical probabilistic information entropy is a commonly used mechanism for a heuristic method construction of decision tree learning. The entropy kernel potentially links its deviation and decision tree classification performance. This paper presents a decision tree learning algorithm based on constrained gain and depth induction optimization. Firstly, the calculation and analysis of single- and multi-value event uncertainty distributions of information entropy is followed by an enhanced property of single-value event entropy kernel and multi-value event entropy peaks as well as a reciprocal relationship between peak location and the number of possible events. Secondly, this study proposed an estimated method for information entropy whose entropy kernel is replaced with a peak-shift sine function to establish a decision tree learning (CGDT) algorithm on the basis of constraint gain. Finally, by combining branch convergence and fan-out indices under an inductive depth of a decision tree, we built a constraint gained and depth inductive improved decision tree (CGDIDT) learning algorithm. Results show the benefits of the CGDT and CGDIDT algorithms.

## 1. Introduction

Decision trees are used extensively in data modelling of a system and rapid real-time prediction for real complex environments [1,2,3,4,5]. Given a dataset acquired by field sampling, a decision attribute is determined through a heuristic method [6,7] for training a decision tree. Considering that the heuristic method is the core of induction to a decision tree, many researchers have contributed substantially to studying an inductive attribute evaluation [8,9,10]. Currently, the heuristic method of attribute selection remains an interesting topic in improving learning deviation.

The attribute selections in constructing a decision tree are mostly based on the uncertainty heuristic method, which can be divided into the following categories: Information entropy method based on statistical probability [11,12,13,14], based on a rough set and its information entropy method [15,16,17], and the uncertainty approximate calculation method [18,19]. An uncertainty evaluation of Shannon information entropy [20] based on statistical probability has been used previously for uncertainty evaluation of the sample set division of decision tree training [21], such as the well-known ID3 and C4.5 heuristic method of the decision tree algorithm [22,23]; these methods are used to search for a gain-optimized splitting feature of dividing subsets for an inductive classification to achieve a rapid convergence effect. Whilst a rough set has the advantage of natural inaccuracy expression, through the dependency evaluation of condition and decision attributes, the kernel characteristics of its strong condition quality is selected as the split attribute to form the decision tree algorithm, with improved classification performance [15,16,17,24]. The uncertainty approximation calculations focus on the existence of the deviation in an evaluation function estimated by most learning methods with information theory [18]. These computations further improve the stability of the algorithm by improving the uncertainty estimation of entropy [25,26,27,28]. The deviation in entropy is not only from itself, but also from the properties of data and samples.

This study proposed an improved learning algorithm based on constraint gain and depth induction for a decision tree. To suppress the deviation of entropy itself, we firstly used a peak-shift sine factor that is embedded in the information entropy to create a constraint gain *GCE* heuristic in accordance with the entropy law of peak, which moves to a low probability and intensity enhancement whilst increasing the number of events possible. The uncertainty is represented moderately so that the estimation deviation could be avoided. This phenomenon realizes the uncertainty estimation considering the otherness of the data property while allowing for entropy itself. Moreover, evaluation indicators of branch inductive convergence and fan-out are used in assisting the heuristic *GCE* to select a minimal attribute that is affected by data samples and noises on the basis of the primary attributes. This study obtained an improved learning algorithm through an uncertainty-optimized estimation for the attributes of a decision tree. The experimental results validate the effectiveness of our proposed method.

The rest of this paper is organized as follows. Section 2 introduces some related works on the attribute selection of heuristic measures in a decision tree. Section 3 discusses the evaluation of uncertainty. Section 4 proposes a learning algorithm based on the constraint gain and optimal depth for a decision tree. Section 5 introduces the experimental setup and results. Section 6 concludes the paper.

## 2. Related Work

Decision tree learning aims to reduce the distribution uncertainty of a dataset, which is partitioned by selected split attributes, and enables a classified model of induction to be simple and reasonable. Notably, a heuristic model based on the uncertainty of entropy evaluation has become a common pattern for decision tree learning given its improved uncertainty interpretation.

The uncertainty evaluation based on information entropy was design by Quinlan in the ID3 algorithm [22] in which the uncertainty entropy of a class distribution, *H*(*C*), is reduced by the class distribution uncertainty, *E*(*A*), of the attribute domain in the dataset, namely *H*(*C*)-*E*(*A*); thus, a heuristic method of information gain with an intuitive interpretation is obtained. Given that *H*(*C*) is a constant of the corresponding dataset, the information gain is the minimum calculation of the *Gain* uncertainty at the core, *E*(*A*). In the attribute domain of *E*(*A*), a small distribution of the classification uncertainty is easily obtained using multi-valued attributes, thereby leading to an evident multi-valued attribute selection bias and splitting of the attribute selection instability in the *Gain* heuristic. The C4.5 algorithm [23] uses an *H*(*A*) entropy to normalise *Gain* in an attempt to suppress the bias of a split attribute selection; furthermore, this algorithm improves the classification performance of a decision tree through a pruning operation. Some authors [14] used a class constraint entropy to calculate the uncertainty of the attribute convergence and achieved an improved attribute selection bias and performance.

Nowozin [18] considered information entropy to be biased and proposed the use of discrete and differential entropy to replace the uncertainty estimation operator of a traditional information entropy. These authors found that improving the predictive performance originates from enhancing the information gain.

Wang et al. [29] introduced the embedding of interest factors in the *Gain* heuristic, *E*(*A*), in which the process is simplified as a division operation of a product, and the sum of the category sample accounts for each attribute to form an improved PVF-ID3 algorithm. According to Nurpratami et al. [30], space entropy denotes that the ratio of the class inner distance to the outer distance is embedded in information entropy. Then, space entropy is utilised, rather than information entropy, to constitute the information gain estimation between the target attribute and support information to achieve hot and non-hot spot heuristic predictions.

Sivakumar et al. [31] proposed to use *Renyi* entropy to replace the information entropy in the *Gain* heuristic. Moreover, the normalisation factor, *V*(*k*), was used to improve the *Gain* instability, thereby improving the performance of the decision tree. Wang et al. [29] suggested to replace the entropy of information gain with unified *Tsallis* entropy and determined the optimal heuristic method through *q* parameter selection. Some authors [32] proposed that the deviation of the Shannon entropy is improved by the sine function-restraining entropy peak, but the impact of the property distribution and data sampling imbalance should be ignored.

In addition, Qiu C et al. [33] introduced a randomly selected decision tree, which aims to keep the high classification accuracy while also reducing the total test cost.

## 3. Measure of Uncertainty

### 3.1. Analysis of Uncertainty Measure of Entropy

The concept of entropy has been previously utilized to measure the degree of disorder in a thermodynamic system. Shannon [20] introduced this thermodynamic entropy into information theory to define the information entropy.

We assume that a random variable, *X_j_*, who has *v* possible occurrence can be obtained from things’ space. If we aimed to measure the heterogeneity of things’ status through *X_j_*, then its measured information entropy is described as follows:

(1) $$H(X_{j})=\sum_{k=1}^{v}P(X_{jk})I(X_{jk})=\sum_{k=1}^{v}-P(X_{jk})\log_{2}P(X_{jk})$$

where *X_j_*∈*A* is a random variable derived from an attribute of things, *A* is the attribute set, and *X_jk_* is an arbitrary value of this attribute. *P*(*X_jk_*) is the occurrence probability of the event represented by this attribute value. *I*(*X_jk_*) is the self-information of *X_jk_*, and *H*(*X_j_*) is a physical quantity that measures the amount of information provided by the random variable, *X_j_*. 

**Figure 1:**
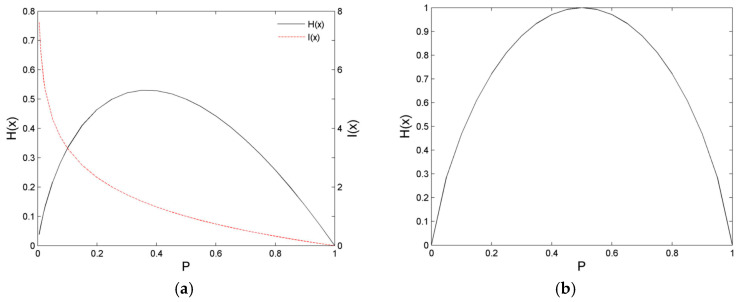
Entropy of single-value event and two-value event. (**a**) Comparison of *I*(*x*) and *H*(*x*) for a single-value event; (**b**) Entropy, *H*(*x*), of two-value event.

**Figure 2:**
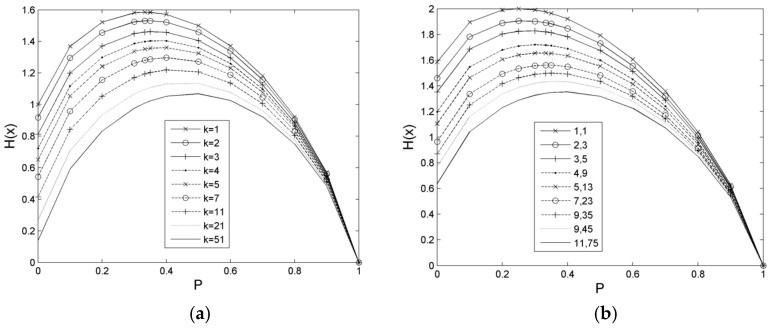
Distribution analysis of multi-value event entropy. (**a**) Entropy of three-value event; (**b**) Entropy of four-value event. Note: (1) If three possible probabilities of the event are *P*(*X_j_*_1_), *P*(*X_j_*_2_), and *P*(*X_j_*_3_), respectively, let parameter *k* exist to make *P*(*X_j_*_3_) = *k P*(*X_j_*_2_), then, *P*(*X_j_*_2_) = [1 − *P*(*X_j_*_1_)]/(1 + *k*) in which *k* > 0. (2) If four possible probabilities of the event are *P*(*X_j_*_1_), *P*(*X_j_*_2_), *P*(*X_j_*_3_), and *P*(*X_j_*_4_), let parameter *k*_1_and *k*_2_ exist to make *P*(*X_j_*_3_) = *k*_1_ *P*(*X_j_*_2_) and *P*(*X_j_*_4_) = *k*_2_ *P*(*X_j_*_2_), then, *P*(*X_j_*_2_) = [1 − *P* (*X_j_*_1_)]/(1 + *k*_1_ + *k*_2_), in which *k*_1_ > 0 and *k*_2_ > 0. Sign “1,1” similar to Figure (**b**), the number at the front is *k*_1_, and the number after it is *k*_2_.

We assume that the number of possible values, *v*, is defined as 1 – 4 for a random variable, *X_j_*. Firstly, the occurrence probability of the event is generated by different parameter settings, and then the distribution of the information entropy is calculated. The results of these calculations are illustrated in Figure 1 and Figure 2. The distribution of the information entropy of the single-value event is an asymmetric convex peak, which changes steeply in a small probability region and slowly in a large probability region. The position of the peak top is at *P* ≈ 0.36788, and the maximum value is *H*_max_(*X_j_*) = 0.53073, as depicted in Figure 1a. The distribution of the information entropy of a double-value event is a symmetric convex peak with probability of *P* = 0.5 whose maximum value is *H*_max_(*X_j_*) = 1, as demonstrated in Figure 1b. The distribution shape of the information entropy of a three-value event. Three-value events are convex peaks with left steep and right slow, as exhibited in Figure 2a,b. The preceding peak top is at *P* = 1/3, and its maximum value is *H*_max_(*X_j_*) ≈ 1.58496. The rear peak top is at *P* = 0.25, and its maximum value is *H*_max_(*X_j_*) = 2.

When the number of values for a random variable is greater than 2, the peak of the information entropy of the random variable not only moves regularly with the change in the values, but also increases its peak intensity to greater than 1. Therefore, the deviation in the uncertainty estimation is protruded by the change in the value number of the random variable, whereas the peak shift of the information entropy presents the uncertainty distribution.

### 3.2. Definition of Constraint Entropy Estimation Based on Peak-Shift

For a random variable, *X_j_* ∈{*X*_1_, *X*_2_*,..., X*_m_}, which represents the attribute to the event that occurred in things’ space, if a reasonable uncertainty evaluation of the *X_j_* variable on all attributes’ set is required, then the effect of the possible number of random variables on the intensity of the information entropy is eliminated. This condition aims to create a normalized measurement of uncertainty. We define the entropy estimation to measure the uncertainty of this random variable as follows:

(2) $$H_{sc}(X_{j})=\sum_{k=1}^{v}P(X_{jk})I_{sin}(X_{jk},v)$$

where *H*_sc_ is the average of the uncertainty of a random variable, *X_j_*. We aim for this variable to be no more than 0.5 when it is a single-value variable. The kernel, *I*_sin_, of the entropy is the key part of deviation improvement, that is, the sine function is used to replace the entropy kernel, *log*_2_*x*^−1^, partly in accordance with this requirement. The peak-shift entropy kernel definition based on the peak intensity constraint is expressed as follows:

(3) $$I_{sin}(X_{jk},v)=Sin(\omega (X_{jk},v)P(X_{jk})+\Psi (X_{jk},v))$$

where *ω*(*X_jk_*, *v*) is a periodic parameter, and *Ψ* (*X_jk_*,*v*) is the initial phase parameter.

**Figure 3:**
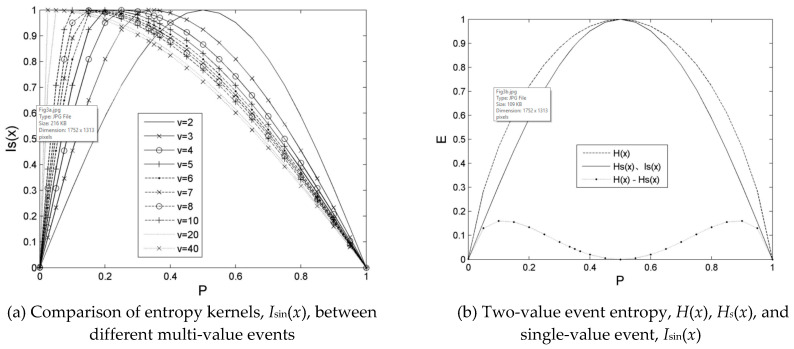
Comparison of *I*_sin_(*x*) for different values events, analysis of *H*(*x*) and *H_s_*(*x*) for two-value event. Note: Is(x) is *I*_sin_(*x*) in the figure.

If *v* = 1, and *P*(*X_jk_*) ∈ [0,1], then *I*_sin_ (*X_jk_*, *v*) is the first half of a single cycle sine function with the initial phase as 0 in the probability [0,1] domain. Its form is expressed in Formula (4), and the distribution of *I*_sin_(*X_jk_*,1) is displayed in Figure 3b:

(4) $$I_{sin}(X_{jk},v)=Sin(\pi \cdot P(X_{jk})/2)$$

If *v >* 1, then *I*_sin_ (*X_jk_*,*v*) is the entropy kernel of the peak top (summit position, *SP*) transferred to *P* = 1/*v*; that is, the kernel refers to the connection composition of two cycles and initial phases, namely it consists of two 1/2 radian period sine function in the probability [0,1] domain.

When *P*(*X_jk_*) ∈ [0, 1/*v*], *I*_s_ (*X_jk_*, *v*) is the monotonically increasing distribution of the first 1/2 radian period sine. The formula is expressed in Formula (5):

(5) $$I_{sin}(X_{jk},v)=Sin(v\pi \cdot P(X_{jk})/2)$$

When *P*(*X_jk_*) ∈ (1/*v*, 1], *I*_sin_ (*X_jk_*, *v*) is the monotonically decreasing distribution of the second 1/2 radian period sine. The formula is defined in Formula (6):

(6) $$I_{sin}(X_{jk},v)=Sin(\frac{v\cdot P(X_{jk})-1}{v-1}\cdot \frac{\pi }{2}+\frac{\pi }{2})$$

The above connection position of the two initial parameterised sinusoids, which constituted *I*_sin_ (*X_jk_*, *v*), is also where the entropy kernel amplitude has the maximum value.

When the number of random variable values, *v*, increases gradually from 2, the entropy kernel, *I*_sin_, summit is transferred gradually from the probability of 0.5 to a small probability, which is presented in Figure 3a. This design aims to enhance the uncertainty expression of the random variable; that is, a resonance at the probability, 1/*v*, because its uncertainty is the largest when all possibilities of the random variable occur with an equal probability.

When *v* = 1, the constraint entropy kernel, *I*_sin_, forms, the impurity is directly harvested in the minimum distribution of extreme probabilities and the maximum of the *P* = 0.5 probability; then, Weibull’s *log*_2_*x*^−1^ kernel forms, the impurity is depicted in the probability product of a monotone decreasing distribution of the first maximum then minimum. The impurity of the constrained entropy kernel is strongly natural.

When *v* = 2, the distribution form is thinner in constraint entropy, *H*_sc_ (*X_j_*), than in conventional entropy, *H*(*X_j_*); that is, *H*_sc_ (*X_j_*) < *H*(*X_j_*) on the left of peak (0, 0.5) and right of peak (0.5, 1.0) with a probability 0.5 symmetry. Their details are plotted in Figure 3b. The difference, *H*(*X_j_*) − *H*_sc_(*X_j_*), between them is evident in the range of the small probability (0, 0.4) and large probability (0.6, 1.0). However, the difference is small in the peak top range (i.e., 0.4, 0.6). Therefore, the constraint entropy, *H*_sc_(*X_j_*), is not only the strongest during an equiprobable occurrence of possibilities for the event, but also presents an amplitude whose suppression is realized at both sides where the probability decreases and then increases, and the influence strength on the uncertainty is reduced.

**Figure 4:**
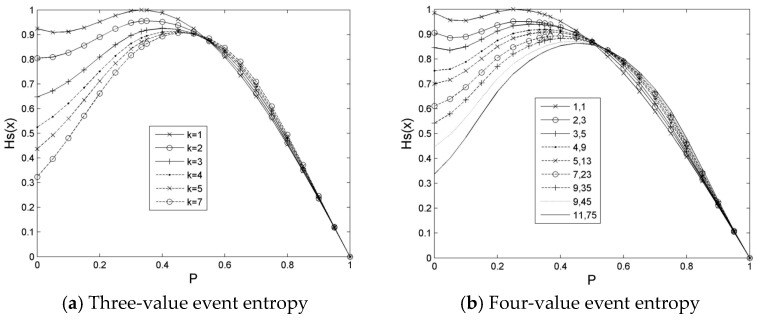
Comparison analysis of multi-value event *H*_s_(*x*) entropy. Note: Same as Figure 2.

When *v* = 3, the random variable has three kinds of possible events to occur whilst a possibility of them shows a range of probability [0,1], and other possibilities may be distributed reversely or randomly. If three kinds of possibilities change to an equal proportion from an unequal proportion, that is, parameter, k = 7, decreases gradually to 1, as depicted in Figure 4a, then the *H*_sc_ (*X_j_*) peak will move to 1/3 probability from 0.5 probability. The right side of the peak changes from steep to gentle, whereas the left side is from gentle to steep. Then, the change gradually increases. Finally, the peak has reached its maximum strength.

**Figure 5:**
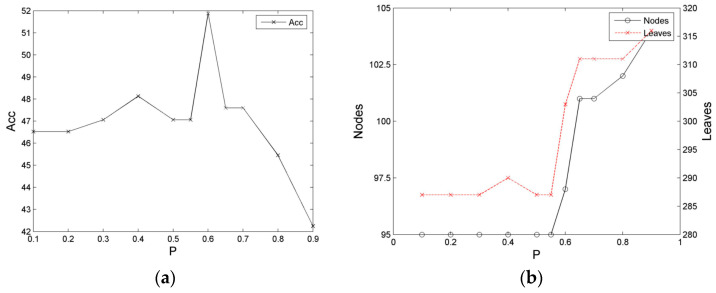
Relationship diagrams of the classification performance of the decision tree of the Balance dataset and the location of the entropy kernel peak. (**a**) Relationship between the accuracy rate and entropy kernel peak location; (**b**) Relationship between the scale and entropy kernel peak location.

When *v* = 4, as possibilities of the event occur from the unequal proportion to equal proportion, that is, when *k*_1_ and *k*_2_ change from large to small, the peak top of *H*_sc_ (*X_j_*) transfers gradually from near P = 0.5 to a small direction until it reaches *P* = 0.25. Finally, the summit value also increases until it reaches the maximum *H*_sc_(0.25) = 1 at *P* = 0.25. The distribution of *H*_sc_(*X_j_*) is demonstrated in Figure 4b, which is similar to Figure 5a. The right side of the peak shows a slow decrease, whereas the left side gradually increases.

In comparison with the traditional information entropy, the constraint entropy estimation, *H*_sc_(*X_j_*), retains firstly the shape and extremal distribution of a peak for an equal proportion of the possible occurrence of a two-value or a multi-value event. Moreover, the constraint entropy estimation restricts the peak intensity of a multi-value event to not more than 1, meanwhile, enhancing the uncertainty of the event in the direction of an equal proportional possible occurrence and weakening the uncertainty of the event in the direction of an unequal proportional possible occurrence. Clearly, although it seems to discard the intuitive expression of information, then the constrained entropy estimation could be more sensitive to discovery uncertainty, reasonable, but not exaggerated.

## 4. Decision Tree Learning Algorithm Based on Constraint Gain and Depth Optimal

### 4.1. Evaluation of the Attribute Selection Based on Constraint Gain and Depth Induction

Considering a node of the decision tree, its corresponding training dataset is *S* = {*Y*_1_, *Y*_2_*, ..., Y_n_*}, in which the attribute variable set of a dataset is {*X*_1_, *X*_2_*, ..., X*_m_}, the class tag of each sample is *T_i_*∈*C,* and *C* is the class set of the things. Thus, each sample, *Y*_i_, consists of the attributes and a class tag, *T_i_*. According to the *Gain* principle [22], we aim to find the attribute of a strong gain in the training dataset, *S*, whilst the impact of attribute otherness is reduced. Therefore, we defined the entropy estimation of gain uncertainty measured on the basis of the peak-shift as follows:

(7) $$GCE(X_{j})=E_{s}(C|X_{j})=\sum_{i=1}^{v}P(X_{ji})H_{sc}(C|X_{ji})$$

where *GCE* is the gain constraint entropy estimation, which is measured by the key changed part from the *Gain* formula, in which the uncertainty measure of the category distribution in the attribute space is *H_sc_*, and *H_sc_* is optimized by the constraint entropy based on the peak-shift. Its specific calculation is expressed in Formula (8):

(8) $$H_{sc}(C|X_{ji})=\sum_{k=1}^{\left|C\right|}P(C_{k}|X_{ji})I_{sin}(P(C_{k}|X_{ji}),v)$$

where *P*(*C_k_*|*X_ji_*) is the probability of distribution of a class, *C_k_*, in the *X_ji_* value domain of an attribute, *I*_sin_(*P*(*C_k_*|*X_ji_*), *v*) is an entropy kernel calculated specifically in accordance with either Formula (5) or (6).

Given the attributes set of the training dataset, *S*, the *GCE* measure is performed in accordance with Formula (7). From this condition, we aim to find an attribute variable of the smallest uncertainty of a class distribution in the attribute space, as defined in Formula (9):

(9) $$A*=\underset{X_{j}\in X}{\arg\min}\{GCE(X_{j})|j\in [1,|X|]\}$$

where A* is a set of candidate attributes that provide a partitioning node, in which the number of values for each attribute is greater than 1.

Whilst evaluating the selection of attributes on the basis of the gain uncertainty formed by the constraint entropy, we must consider the inductive convergence of the branches generated by a selected split attribute to reduce the effects of samples and noise. We assume that the attribute, *X_j_*, is selected as a splitting attribute of the node for the decision tree induction. Given that the attribute’s, *X_j_*, value distribution is {*X_j_*_1_, *X_j_*_2_, ..., *X_jv_*}, the dataset, *S*, is divided into *v* subsets and downward *v* branches. Correspondingly, when an attribute, *X_k_* (*X_k_ ≠ X_j_*), is selected as a further split attribute in the subset of the branches, the convergence branching number under the depth generated by the current tree node attribute, *X_j_*, is measured as follows:

(10) $$B_{conv}(X_{j})=\sum_{l\in V}^{}F_{l}(X_{jl})+\sum_{i\in U}^{}\sum_{q=1}^{kv}F_{q}(X_{kq})$$

where *V* is a set of branch sequence numbers that can be converged as the leaf by the attribute, *X_j_*, and *U* is a set of branch sequence numbers that can be divided further into nodes by an attribute, *X_j_*. By contrast, *X_jl_* and *X_kq_* (*q*∈[0, *kv*], *kv* is the number of *X_k_* values) are the attribute values of the current tree node and the sub-branch node, correspondingly. *F_l_* is the functions that determine whether the branches of the attribute value of the current tree node is a leaf, and *F*_q_ is the functions that determine whether the branches of the attribute value of the sub-branch node are a leaf. If *P*(*C_b_*|*X_jl_*) = 1, and *P*(*C_b_*|*X_kq_*) = 1, where *b*∈[1, |*C*|], then, *F_l_* and *F_q_* are 1; otherwise, 0. Thus, *B*_conv_(*X_j_*) is the strength index, which measures the convergence of a branch at two inductive depths generated by the split attribute, *X_j_*, of the current tree node.

Similarly, when we select an attribute, *X_j_*, as a split attribute of the current node for the decision tree, we can expect to estimate the divergence of branches produced in-depth by *X_j_*. Therefore, the split attribute of a branch node generated by the division of attribute *X_j_* is assumed to be *X_k_* (*X_k_≠X_j_*). Then, the number of fan-outs under the depth generated by the current tree node attribute, *X_j_*, is measured as follows:

(11) $$B_{diver}(X_{j})=N_{j}(X_{j})+\sum_{i=1}^{v}\sum_{q=1}^{kv}N_{k}(X_{k})$$

where *N_j_* is the number of branches generated by the current tree node, and *N_k_* is the number of branches generated by the subordinate node of a branch of the current tree node. Thus, *B_diver_*(*X_j_*) is the aided index that measures the divergence of the branch at the two inductive depths produced by the splitting attribute, *X_j_*, of the current tree node.

### 4.2. Learning Algorithm Based on Constraint Gain and Depth Induction for a Decision Tree

According to the Hunt principle and the above-mentioned definition, this study proposed an inductive system that is the heuristic framework of an optimal measure of a category convergence in the attribute space. In this attribute space, minimal uncertainty distribution is searched based on the constraint mechanism of the strength and summit, and constitutes the decision tree learning algorithm (CGDT) of the constrained gain heuristic. Moreover, whilst *GCE* is used as the main measurement index, the branch convergence, *B_conv_*, and branch fan-out, *B_diver_*, are applied to be auxiliary indices among the similar attributes of *GCE*. We aim to select split attributes of a strong deep convergence and weak divergent, and form the constraint gained and depth inductive improved decision tree learning algorithm (CGDIDT).

Therefore, the learning algorithm based on the constraint entropy for the decision tree designed is defined specifically as follows (Algorithms 1 and 2):


**Algorithm 1. The learning algorithm of the constraint gained decision tree, CGDT (*S*, *R*).**
Input: Training dataset, *S*, which has been filtered and labelled.Output: Output decision tree classifier. Pre-processing: For any sample in the dataset, {*Y*_1_, *Y*_2_*, ..., Y_n_*}: *Y*_i_ = {*X, T_i_*}, *T*_i_∈*C* to obtain the discrete training set.Initialization: The training set, *S*, is used as the initial dataset of the decision tree to establish the root node, *R*, which corresponds to the tree.1. If *Leaftype*(*S*) = *C_k_*, where *C_k_*∈*C* and *k*∈[0, |*C*|], then label the corresponding node, *R*, of the sample set, *S*, as a leaf of the *C_k_* category, and return.2. Return the valid attribute set of the corresponding dataset, *S*, of the node: *X_e_ = Effective*(*S*). If *X_e_* is an empty set, then the maximal frequentness class is taken from the *S* set, and the node is marked as a leaf and is returned. If *X_e_* is only a single attribute set, then this attribute is returned directly as the split attribute of the node.3. For any attribute, *X*_i_ (i∈[0, |*X_e_*|]), in the *X_e_* set, perform calculations on *GCE*. The attribute of the minimum uncertainty is selected as the split attribute, A*, of the current node, *R*.4. The dataset, *S*, of the current node, *R*, can be divided into *v* subsets, {*S*_1_, *S*_2_..., *S_v_*}, which correspond to the attribute values, {*A*_1_, *A*_2_, ..., *A_v_*} of A*.5. For i = 1, 2, ..., *v*
v CGDT(*S*_i_, *R*_i_).

In the algorithm presented above, *Leaftype*(*S*) is the function of a leaf class judgment, and *Effective*(*S*) is the processing function to obtain a valid attribute set of the dataset, *S*. The complexity of the entire CGDT(*S*, *R*) is the same as that of the ID3 algorithm. The core of the algorithm is the attribute selection heuristic algorithm based on *GCE*.


**Algorithm 2. Constraint gained and depth inductive decision tree algorithm, CGDIDT (*S*, *R*).**
Input: Training dataset, *S*, which has been filtered and labelled.Output: Output decision tree classifier. Pre-processing: As Algorithm 1.Initialization: As Algorithm 1.1. Judge whether the *Leaftype*(*S*) = *C_k_* (*C_k_* and *S* definition is the same as in Algorithm 1), the corresponding node, *R*, of the sample set, *S*, is labelled as a leaf of the *C_k_* category when it is true, and return.2. Return the valid attribute set of the corresponding dataset, *S*, of the node: *X_e_ = Effective*(*S*). If *X_e_* is an empty set, then the maximum frequency class is taken from the *S* set. The node is marked as a leaf and is returned. If *X_e_* is only a single attribute set, then return the attribute directly as the split attribute of the node, *R*.3. Establish an empty set, *H*, for the candidate split attributes; firstly, obtain the attribute with the smallest constraint gain, *f*, from the set, *S*, that is, *f* = Min{*GCE*(*X*_i_), i∈[0, |*X_e_|*]}. Secondly, determine the candidate attributes in which *GCE* is the same or similar to the minimum value, such as *GCE*≤(1 + *r*)*f*, where *r*∈[0, 0.5]; these candidate attributes are placed in the set, *H*.4. Face the candidate attributes set, *H*, of the current node, and calculate the depth branch convergence number, *B*_conv_, and depth branch fan-out number, *B*_diver_, of each attribute. If the attribute with the optimal *B*_conv_ is not the same as the *GCE* minimal attribute in the set, *H*, then select the attribute of the larger *B*_conv_ and smaller *B*_diver_ as the improved attribute. If the attribute obtained the optimal *B*_conv_, and the *GCE* minimal attribute is the same attribute in the set, *H*, then the split attribute, A*, selection is all with the *GCE* minimum evaluation as the preferred attribute selection criteria for the current node and even the subsequent branch node.5. Divide the dataset, *S*, of the current node, *R*, into *v* subsets, {*S*_1_, *S*_2_..., *S_v_*}, which correspond to the attribute values, {*A*_1_, *A*_2_, ..., *A_v_*} of A*.6. For i = 1, 2, ..., *v*
CGDIDT(*S*_i_, *R*_i_).

The pruning of the above algorithms is turned off. The branch convergence and fan-out index under the depth are introduced to optimize the learning process of the decision tree on the basis of Algorithm 1.

## 5. Experiment Results

### 5.1. Experimental Setup

In this section, we use the 11 discretized and complete datasets of the UCI international machine learning database as the original sample sets to verify the performance of the CGDT and CGDIDT algorithms. The details of the datasets are provided in Table 1.

Firstly, the representative Balance, Tic-Tac-Toe and Dermatology datasets were selected for the peak shift experiment to observe the effects of the peak movement of the entropy core on decision tree learning. For the first two datasets, the numbers of the attribute values are 5 and 3, respectively, in which their numbers of the attributes values are the same in each dataset. For the subsequent dataset, the numbers of the attributes values are mostly 4, except for two attributes for which the numbers of the values are 2 and 3. The category distributions are Balance: {217, 39, 182}, Tic-Tac-Toe: {439, 232}, and Dermatology: {74, 43, 53, 35, 39, 12}. Regardless of whether from the distribution of the number of attributes values or the distribution of the sample categories, these three datasets are highly representative for the peak shift effect experiment of the constraint entropy. The similar details of other datasets are shown as Table 1.

Then, the CGDT and CGDIDT algorithm experiments were performed separately on the 11 datasets using the experimental system designed in this study. However, the ID3 and C4.5 (J48) decision tree algorithms were implemented as references by the Weka system. The same training and test sets were used for the experiment on different algorithms when the same dataset experiments were performed on two different systems. Before the experiment, the dataset was sampled uniformly and unrepeatably in accordance with the determined proportion, α, in which the extracted parts constituted the training set and the remaining parts constituted the test set for learning, training, and validation. In this study, a sampled proportion of α = 70% was first used for the training set. Even for the Monks datasets, which provided the training sets, this learning experiment still used only α proportional extracted datasets from the provided training set as learning training sets to verify the adaptability of the learning algorithm, whereas all the remaining datasets were used for testing.

The classifier scale (*Size*) of a decision tree on the training set, the accuracy (*Acc*) for verifying the test set, the *F-*measure, and the test coverage (*Cov*) were the indicators used to compare and evaluate the algorithms in the experiment. The description of the specific indicators is given Equations (12) and (13):

(12) $$A\mathrm{cc}=\frac{n_{c}}{n_{t}}\cdot 100\%,\;Size=Ns+Ls$$

where *n_c_* is the number of samples that have been validated in the test set, *n_t_* is the total number of tested samples, *Ns* is the number of nodes of the decision tree that are learnt and obtained in the training set, and *Ls* is the number of leaves of the decision tree that are learnt and obtained in the training set.

(13) $$\text{F-measure}=\frac{2Acc\cdot precision}{Acc+precision},\;Cov=\frac{n_{t}-n_{f}}{n_{t}},\;precision=\frac{n_{c}}{n_{t}-n_{f}}$$

where *n_f_* is the number of samples that tested unsuccessfully in the test set, and *precision* is the degree of accuracy of a test set except to testing failures.

### 5.2. Influence of the Entropy Peak Shift to Decision Tree Learning

**Figure 6:**
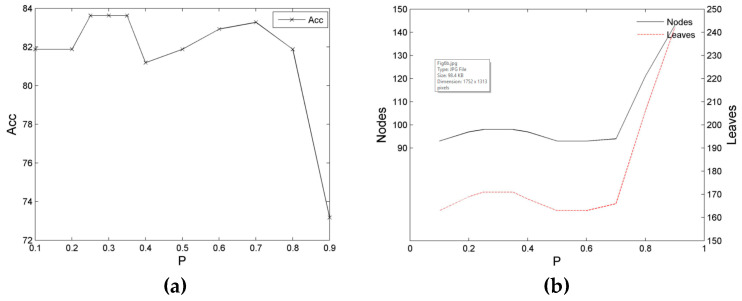
Relationship diagrams of the classification performance of the decision tree of the Tic-tac-toe dataset and the location of the entropy kernel peak. (**a**) Relationship between the accuracy rate and entropy kernel peak location; (**b**) Relationship between the scale and entropy kernel peak location.

**Figure 7:**
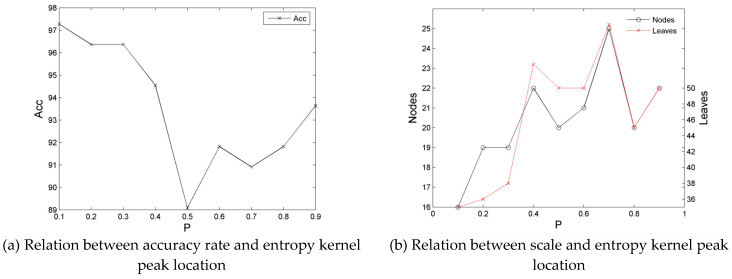
Relationship diagrams of the classification performance of the decision tree of the Dermatology dataset and the location of entropy kernel peak.

In this study, we initially conducted an experiment on the influence of the peak shift of the constraint entropy on decision tree learning. The experiment used the training and test sets of Balance, Tic-Tac-Toe, and Dermatology. The experiment result is presented in Figure 5, Figure 6 and Figure 7.

For the Balance training set, the training of a decision tree is implemented by moving the peak of the constraint entropy as the probability ranges from low to high for the heuristic, namely, *SP*∈[0.1, 0.9]. The accuracy rate of the classification, *Acc*, exhibits a tendency of initially increase and then decrease. Amongst the areas, *Acc* presents a high distribution at section [0.1, 0.7] and a sudden low distribution at section [0.8, 0.9] until it reaches the lowest value, in which *Acc* is relatively steady at section [0.1, 0.55], but appears to be protruding partially at sections [0.35, 0.45] and [0.575, 0.625]. In addition, the numbers of nodes and leaves of the decision tree (*Size*) are relatively low at section [0.1, 0.55] and high at section [0.6, 0.9] until the highest distribution is reached. With the exception of the strong local expressions at sections [0.35, 0.45] and [0.575, 0.625], the numbers of nodes and leaves in other regions exhibit the opposite change distribution.

Figure 6 illustrates the training case of the Tic-tac-toe dataset. The general form of the decision tree, *Acc*, exhibits an initially high and then low distribution; that is, it presents a relatively high distribution at section [0.1, 0.7], in which it maintains a period of high value at section [0.25, 0.35], and then demonstrates a low distribution at section [0.8, 0.9], with a steep decline at section > 0.8. However, the numbers of nodes and leaves of the decision tree (*Size*) exhibit a stable low-value distribution at section [0.1, 0.7], which indicates an improved reverse distribution with *Acc*.

Similarly, for the Dermatology training set, the general form of the decision tree, *Acc*, also displays an initially high and then low distribution; that is, it first achieves a relatively high distribution at section [0.1, 0.3], and then exhibits a low distribution and an apparent hollow bucket shape at section [0.4, 0.9], in which section [0.1, 0.3] is the stable high section of *Acc*, whereas section [0.45, 0.55] is the lowest section. Simultaneously, the numbers of nodes and leaves of a decision tree (*Size*) display a considerably reverse distribution with *Acc*, which is low at section [0.1, 0.3], but high at section [0.4, 0.9]. However, a stronger volatility is achieved, which is lowest at a low *SP* section and highest at a high *SP* section.

The preceding analysis implies that although the Balance and Tic-tac-toe training sets with the same number of attribute values exhibit a better stability distribution of classification performance and size than the Dermatology training set, which has a different number of attribute values, they all have the same volatility and regularity is evident. That is, the Balance set at section [0.1, 0.3], Tic-tac-toe set at section [0.25, 0.35], and Dermatology set at section [0.1, 0.3] demonstrate improved agreement in terms of the accuracy rate with the numbers of nodes and leaves. The details of which are as follows:

(1) For the Tic-tac-toe set with the same number of attribute values and a similar proportion of sample categories, the accuracy rate and size (numbers of nodes and leaves) exhibit the best expression for the decision tree when the peak of the entropy core, *SP*, is constrained at *P* = 1/3.

(2) For the Balance set with the same number of attribute values and a slightly different proportion of sample categories, the corresponding numbers of nodes and leaves present the most stable expression when SP is constrained at *P* = 1/5, although the accuracy rate of the decision tree does not reach the maximum value.

(3) For the Dermatology set with different attribute values and a similar difference in the proportion of samples categories, the corresponding numbers of nodes and leaves are small when *SP* is constrained at *P* ≤ 1/4, whereas the accuracy rate of the decision tree is high.

The experiment on the three representative datasets shows that uncertainty is measured by the constraint entropy, which consists of the dynamic peak shift of the entropy core in accordance with *SP* = 1/*v*. Enhancing the rationality of split attribute selection is effective for decision tree induction.

### 5.3. Effects of Decision Tree Learning Based on Constraint Gain

In accordance with the rules obtained from the preceding experiment on the peak shift of the entropy core, the constraint entropy of improved dynamic peak localization is determined. Its contribution constitutes the *GCE* heuristic and it realizes the decision tree algorithm based on constraint gain heuristic learning, CGDT. In this section, 11 datasets were used to compare the *Gain* and *Gainratio* heuristics (pruning off). The experimental results are presented in Table 2.

Firstly, the classification results of the decision tree are observed. The classification accuracy rate, *Acc*, of the *GCE* heuristic is better than those of the *Gain* and *Gainratio* heuristics in eight and seven datasets, respectively. The difference range of the former was 0.6494–6.6667%, and the mean value was 2.7464. That of the latter was 0.4762–23.6363%, and the mean value was 8.2477. In other datasets, *GCE* has two datasets with the same *Acc* as that of the *Gain* heuristic, and another dataset with a weaker *Acc* than that of the *Gain* heuristic, but stronger than that of the *Gainratio* heuristic. The difference range is 0%—−1.905%, and the mean is −0.605. Meanwhile, *GCE* has one dataset with the same *Acc* as that of the *Gainratio* heuristic, whereas the other three datasets are poor. The difference range is 0%–−4.8701%, and the mean is −2.5373. Accordingly, the classification *Acc* of only four datasets of the *Gainratio* heuristic are better than that of the *Gain* heuristic.

Secondly, the size of the decision tree classifier is observed. The *GCE* heuristic has five datasets with a smaller decision tree classifier than that of the *Gain* heuristic and nine datasets with a smaller decision tree classifier than that of the *Gainratio* heuristic. The decision tree classifiers of the other datasets are the same or close to one another. The *Gainratio* heuristic has two datasets with smaller decision tree classifiers than those of the *Gain* heuristic. The other datasets have decision tree classifiers that are considerably larger than those of the *Gain* heuristic.

For the larger Mushroom dataset, the learning experiment of reducing the sampling proportion to 50% was conducted. The classification accuracy, *Acc*, of the *GCE* heuristic is better than those of the *Gain* and *Gainratio* heuristics. The size of the decision tree classifier is larger than that of the *Gain* heuristics and smaller than that of the *Gainratio* heuristic.

From the overall average of all the datasets, the numbers of branch nodes (30.6364) and leaves (70) of the *GCE* heuristic are extremely close to those of the *Gain* heuristic (29.9091, 69) and considerably less than those of the *Gainratio* heuristic. Meanwhile, the average size of the *GCE* heuristic’s decision tree classifier is close to that of the *Gain* heuristic. The average accuracy of the *GCE* heuristic (82.6265) is better than those of the *Gain* and *Gainratio* heuristics (80.8023 and 78.3007, respectively).

On average, the *GCE* heuristic based on the constraint entropy for a decision tree achieves better classification accuracy than the *Gain* and *Gainratio* heuristics.

### 5.4. Effect of Optimized Learning of Combining Depth Induction

In the preceding section, the classifier for a decision tree is established through the inspired learning of *GCE* in the CGDT algorithm. Its size characteristics show that the split attribute of tree nodes should still be optimized in inductive convergence. CGDIDT is a learning algorithm of deep induction optimization that is based on the *GCE* selection for a decision tree. It is compared with ID3 and J48 of the Weka system. The experimental results are presented in Table 3.

The classification results of the CGDIDT decision tree demonstrate that the accuracy of 10 datasets is greater than that of the ID3 algorithm, with differences ranging from 1.4085 to 14.6104. Meanwhile, one dataset is flat and the average difference is 4.7312. The size of nine datasets is less than that of the ID3 algorithm, with a difference of −1–−104. The *F*-measure has 10 datasets that are greater than the ID3 algorithm. Its coverage has eight datasets that are greater than the ID3 algorithm.

CGDIDT is also compared with the J48 algorithm. Its accuracy has five datasets that are better than the average difference (8.3968), one dataset is flat and five datasets are weak (with an average difference of −5.2556). Their average overall difference is 1.4278. Meanwhile, the size of 10 datasets is bigger than that of J48. The *F*-measure has six better datasets, and its coverage has seven smaller datasets and three flat datasets.

Finally, the J48 algorithm is compared with ID3. The accuracy has seven better datasets, one flat dataset and five weak datasets. The average difference is 3.2854. Moreover, size has 11 smaller datasets. The *F*-measure has seven better datasets, and its coverage has eight smaller datasets.

For the larger Mushroom dataset, the experimental results of reducing the sampling proportion to 50% are as follows: The classification performance (*Acc* and *F*-measure) of the CGDIDT algorithm is better than that of ID3 and the same as that of J48. Meanwhile, the size of CGDIDT is smaller than those of ID3 and J48.

In conclusion, the classification accuracy and *F*-measure of the CGDIDT algorithm are averagely better than those of the ID3 and J48 algorithms of the Weka system. The average classification performance is further improved compared with that of CGDT. The average size and coverage of the classifier is considerably improved compared with those of ID3. However, the classifier scale is weaker than that of the J48 algorithm, which is the reason why the built-in pruning of J48 plays an evident role in the Weka system.

## 6. Conclusions

This study proposed an optimal learning algorithm based on the constraint gain for a decision tree. This study firstly analyzed the uncertainty distributions of single-event and multi-event entropies in accordance with the composition of information entropy. It found an enhanced property of the peak entropy value with a number of events and the existence of a relationship between the peak position and the reciprocal number of events. Hence, by replacing the information entropy kernel with the peak shift sine to achieve the uncertainty-estimated entropy of enhanced restraining, we proposed an attribute selection heuristic based on constraint gain to obtain the learning algorithm, CGDT. Then, we built an optimal learning algorithm, CGDIDT, using the branch convergence and fan-out indices within the inductive depth of a decision tree to assist in the selection optimization of the split attribute for a decision tree. The comparison of the experimental results showed that the classification accuracy of a decision tree based on the *GCE* heuristic is averagely superior to those of *Gain* and *Gainratio*. The size of the *GCE* heuristic is close that of *Gain* and larger than that of *Gainratio*. Finally, the average *Acc* and *F*-measure of the proposed CGDIDT algorithm are superior to those of ID3 and J48, whereas its size is generally smaller than that of ID3, but larger than that of J48. 

For the classifier size of the decision tree, the CGDIDT was weaker than the pruned algorithm although it was better improved than ID3. This should be a need for future research and improvement.

## Figures and Tables

**Table 1:** Experimental datasets from the UCI machine learning repository.

No.	Dataset	Instances	Attributes	Num (A.v.) Range	Distribution of Attributes (*n*/*v*)	Num. of Class values Distr. of Class
1	Balance Scale	625	4	5~5	4/5	3{288,49,288}
2	Breast	699	9	9~11	1/9, 7/10, 1/11	2{458,261}
3	Dermatology	366	33	2~4	1/(2,3), 31/4	6{112,61,72,49,52,20}
4	Tic-Tac-Toe	958	9	3~3	9/3	2{626,332}
5	Voting	232	16	2~2	16/2	2{124,108}
6	Mushroom	8124	22	1~12	1/(1,7,10,12), 5/2, 4/4, 2/5, 2/6, 3/9	2{4208,3916}
7	Promoters	106	57	4~4	57/4	2{53,53}
8	Zoo	101	18	2~6	15/2, 1/6	7{41,20,5,13,4,8,10}
9	Monks1 *	124+308	6	2~4	2/2, 3/3, 1/4	2{62,62}
10	Monks2 *	169+263	6	2~4	2/2, 3/3, 1/4	2{105,64}
11	Monks3 *	122+310	6	2~4	2/2, 3/3, 1/4	2{62,60}

Note: Sign “Num(A.v.) range” denotes range of number of attribute values, “{ }” denotes the number of samples for a class. Sign “n/*v*” denotes the number *n* of attributes for same values number *v*. Sign * denoted datasets are training datasets of Monk as the original samples set.

**Table 2:** The experimental results of decision tree Learning based on constraint gain.

Dataset	Method	Size	Ns	Ls	Acc
Balance Scale	Gain	388	97	291	43.8503
	Gainratio	393	98	295	43.8503
	GCE (CGDT)	382	95	287	46.5241
Breast	Gain	78	13	65	89.0476
	Gainratio	107	19	88	86.6667
	GCE (CGDT)	87	16	71	87.1429
Dermatology	Gain	64	20	44	93.6364
	Gainratio	159	51	108	72.7273
	GCE (CGDT)	58	20	38	96.3636
Tic-Tac-Toe	Gain	255	92	163	81.1847
	Gainratio	418	157	261	69.6864
	GCE (CGDT)	269	98	171	83.6237
Voting	Gain	27	13	14	94.3662
	Gainratio	29	14	15	97.1831
	GCE (CGDT)	27	13	14	95.7747
Mushroom	Gain	29	5	24	100
	Gainratio	45	9	36	100
	GCE (CGDT)	35	6	29	100
Mushroom **	Gain	30	5	25	99.8031
	Gainratio	48	9	39	99.8031
	GCE (CGDT)	34	6	28	99.9508
Promoters	Gain	30	8	22	71.8750
	Gainratio	30	8	22	68.7500
	GCE (CGDT)	29	8	21	75.0000
Zoo	Gain	23	9	14	93.3333
	Gainratio	23	9	14	90.0000
	GCE (CGDT)	19	7	12	100
Monks1 *	Gain	58	21	37	77.5974
	Gainratio	52	19	33	83.1169
	GCE (CGDT)	58	21	37	78.2468
Monks2 *	Gain	109	43	66	53.6122
	Gainratio	116	48	68	55.1331
	GCE (CGDT)	108	42	66	55.8935
Monks3 *	Gain	27	11	19	90.3226
	Gainratio	25	11	21	94.1935
	GCE (CGDT)	35	11	19	90.3226
	Gain	98.9091	29.9091	69	80.8023
Average	Gainratio	127	40	87	78.3007
	GCE (CGDT)	100.6364	30.6364	70	82.6265

Note: The sign ‘**’ denotes the sampling proportion α = 50%, and its results are not considered in the average calculation. The sign ‘*’ is the same of Table 1.

**Table 3:** The experimental results of optimized learning based on constraint gain for the decision tree.

Dataset	Method	Size	Acc	Cov	F-Measure
Balance Scale	ID3	486	43.8503	47.5936	0.5942
	J48(C4.5)	21	67.9144	100	0.6791
	CGDIDT	382	46.5241	49.7326	0.6214
Breast	ID3	135	89.0476	94.7619	0.9144
	J48(C4.5)	32	92.8571	100	0.9286
	CGDIDT	100	91.4286	97.1429	0.9275
Dermatology	ID3	81	93.5780	98.1651	0.9444
	J48(C4.5)	25	95.4128	100	0.9541
	CGDIDT	66	98.1818	100	0.9818
Tic-Tac-Toe	ID3	276	81.1847	97.2125	0.8233
	J48(C4.5)	100	83.9721	100	0.8397
	CGDIDT	243	87.4564	97.9094	0.8838
Voting	ID3	27	94.3662	100	0.9437
	J48(C4.5)	3	97.1831	100	0.9718
	CGDIDT	23	95.7747	100	0.9577
Mushroom	ID3	38	100	100	1.0000
	J48(C4.5)	31	100	100	1.0000
	CGDIDT	28	100	100	1.0000
Mushroom **	ID3	34	99.8031	100	0.9980
	J48(C4.5)	29	100	100	1.0000
	CGDIDT	27	100	100	1.0000
Promoters	ID3	33	71.8750	96.8750	0.7302
	J48(C4.5)	17	68.7500	100	0.6875
	CGDIDT	32	75.0000	96.8750	0.7619
Zoo	ID3	23	93.3333	93.3333	0.9655
	J48(C4.5)	17	90.0000	90.0000	0.9474
	CGDIDT	19	100	100	1.0000
Monks1 *	ID3	64	77.5974	88.6364	0.8227
	J48(C4.5)	10	72.7273	100	0.7273
	CGDIDT	39	92.2078	92.2078	0.9595
Monks2 *	ID3	103	51.3308	95.4373	0.5253
	J48(C4.5)	22	57.0342	100	0.5703
	CGDIDT	108	56.2738	96.1977	0.5736
Monks3 *	ID3	32	90.3226	94.1935	0.9302
	J48(C4.5)	12	96.7742	100	0.9677
	CGDIDT	32	95.4839	98.7097	0.9610
	ID3	118	80.5896	91.4735	0.8358
Average	J48(C4.5)	26.3636	83.8750	99.0909	0.8430
	CGDIDT	97.4545	85.3028	93.5250	0.8753

Note: The sign ‘**’ denotes the sampling proportion α = 50%, and its results are not considered in the average calculation. The sign ‘*’ is the same of Table 1.
